# Apoptosis of resident and inflammatory macrophages before and during the inflammatory response of the virgin bovine mammary gland

**DOI:** 10.1186/1751-0147-52-12

**Published:** 2010-02-09

**Authors:** Zbysek Sladek, Dusan Rysanek

**Affiliations:** 1 Department of Morphology, Physiology and Animal Genetics, Mendel University, Zemedelska 1, 613 00 Brno, Czech Republic; 2 Veterinary Research Institute, Hudcova 70, 621 32 Brno, Czech Republic

## Abstract

**Background:**

Macrophages may play a prominent role in defense of the bovine mammary gland, and their functionality is necessary for successful eradication of bacterial pathogens. In contrast to necrosis, however, apoptosis has not yet been studied in macrophages from bovine mammary glands. Therefore, the aim of this study was to confirm the occurrence of apoptosis in macrophages from resting heifer mammary glands and during the inflammatory response.

**Methods:**

Inflammatory response was induced by phosphate buffered saline (PBS) and by lipopolysaccharide (LPS). Resident macrophages (_RES_MAC) were obtained before and inflammatory macrophages (_INF_MAC) 24, 48, 72 and 168 hours after inducing inflammatory response in mammary glands of unbred heifers. Cell samples were analyzed for differential counts, apoptosis and necrosis using flow cytometry.

**Results:**

Populations of _RES_MAC and _INF_MAC contained monocyte-like cells and vacuolized cells. Apoptosis was detected differentially in both morphologically different types of _RES_MAC and _INF_MAC and also during initiation and resolution of the inflammatory response. In the _RES_MAC population, approximately one-tenth of monocyte-like cells and one-third of vacuolized cells were apoptotic. In the _INF_MAC population obtained 24 h after PBS treatment, approximately one-tenth of monocyte-like cells and almost one-quarter of vacuolized cells were apoptotic. At the same time following LPS, however, we observed a significantly lower percentage of apoptotic cells in the population of monocyte-like _INF_MAC and vacuolized _INF_MAC. Moreover, a higher percentage of apoptotic cells in _INF_MAC was detected during all time points after PBS in contrast to LPS. Comparing _RES_MAC and _INF_MAC, we observed that vacuolized cells from populations of _RES_MAC and _INF_MAC underwent apoptosis more intensively than did monocyte-like cells.

**Conclusions:**

We conclude that apoptosis of virgin mammary gland macrophages is involved in regulating their lifespan, and it is involved in the resolution process of the inflammatory response.

## Background

Heifers' mammary glands are susceptible to bacterial infections just as are the lactating and non-lactating mammary glands of cows. Intramammary quarter infection occurrence is very high and may reach nearly 75% in the prepartum period of heifers [1]. The prevalence of intramammary infections in heifers around calving time is very high as well. Unfortunately, little information is available about these infections' relevance for the heifers and relation to post-partum clinical mastitis [2].

A cellular defense system is present in the heifer mammary gland to resist invading bacteria, and it includes macrophages, lymphocytes, and polymorphonuclear leukocytes (PMN) [3-5]. This resident cell population is an early sensor of infection and initiates the immune response following pathogen entry through the teat canal. It is especially important in virgin mammary gland, because there is no cell renewal due to regular milking as in the lactating mammary gland of cows.

Once invading pathogens are detected, the resident macrophages release cytokines, eicosanoids, acute phase proteins and chemoattractants that direct migration of PMN from the blood into the infected area [6,7]. The influx of PMN is followed by the infiltration from the bloodstream of monocytes, which mature locally into inflammatory macrophages [3] and phagocytose bacteria together with PMN [8]. Once the initiating noxious materials are removed via phagocytosis, the inflammatory reaction must still be resolved. Therefore, PMN undergo apoptosis (programmed cell death) and are subsequently phagocytosed by macrophages in bovine mammary glands [9-11].

It is evident that macrophages play a critical role in the initiation, maintenance, and resolution of inflammation [12]. Nevertheless, the high incidence of intramammary infection suggests that the heifer mammary gland's defense system fails to prevent bacterial infections [13]. Macrophages, as a dominant cell type, may be responsible for this situation. Even though these were discovered many years ago [3,14], very little is known about the basic biological features of macrophages in heifer mammary glands.

It is generally known that macrophages are long-lived cells that may persist in the non-inflamed tissues for weeks [15]. Throughout this period, and until they re-emigrate into supramammary lymphatic nodes [16], macrophages need to retain viability if they are to function fully. Not all macrophages can re-emigrate, however, and part of them die by necrosis inside of the mammary gland [17].

It has been observed that in addition to necrosis macrophages also undergo apoptosis locally. Apoptosis has been observed in alveolar, peritoneal and pleural macrophages in response to pathogenic and non-pathogenic stimuli, and a number of mechanisms are recognized as driving this process [18].

It is surprising that little is yet known about macrophage apoptosis in bovine veterinary medicine [19-22] in comparison to human medicine. Furthermore, in contrast to PMN [9-11] and to lymphocytes [23], apoptosis has not yet been studied in macrophages from bovine mammary glands. Therefore, it remains unclear whether apoptosis is involved in regulating the lifespan in macrophages and whether apoptosis of these cells participates in the inflammatory response of the virgin mammary gland.

The aim of this study, therefore, was to confirm the occurrence of apoptosis in macrophages from resting heifer mammary glands and during an inflammatory response. For this purpose, an inflammation model based on induction of inflammatory response in mammary glands of virgin heifers was used. We analyzed two different populations of macrophages - resident macrophages obtained from intact mammary glands, and inflammatory macrophages - through an experimental inflammatory response induced by bacterial and nonbacterial agents.

## Methods

### Animals

The experiments were carried out on 40 mammary glands of 10 virgin, clinically healthy Holstein × Bohemian Pied crossbred heifers aged 15 to 20 months. The heifers were housed in an experimental tie-stall barn and fed a standard ration consisting of hay and concentrates with mineral supplements. The experimental tie-stall used in this study is certified. The animal care conformed to good care practice protocol. All experimental procedures were approved by the Central Commission for Animal Welfare of the Czech Republic. All heifers were free of intramammary infections, as demonstrated by bacteriological examination of mammary lavages.

### Experimental design

Two populations of macrophages were studied: the population of resident macrophages and the population of inflammatory macrophages obtained before and after inflammatory induction of mammary glands respectively, using phosphate buffered saline (PBS) and lipopolysaccharide (LPS). After inflammatory induction, macrophages were collected at four time points (24, 48, 72 and 168 hours). In the fresh cell populations obtained, the total cell count was assessed by the fluoro-opto-electronic method. The differential leukocyte count and number of apoptotic and necrotic cells were detected by flow cytometry (FCM). Moreover, the cells were cultivated *in vitro *and thereafter apoptosis, necrosis, CD11b and CD14 expressions were analyzed by FCM. Finally, cytolysis was assessed by ELISA l actate dehydrogenase (LDH) determination.

### Isolation of resident and inflammatory macrophages

The untreated mammary glands of virgin heifers were rinsed with PBS to obtain the cell population. The population was composed of the resident cells from the mammary glands, which had never before been rinsed. This population of macrophages was designated as resident macrophages (_RES_MAC). The procedure has been used and described many times in our previous studies [24,25]. Briefly, modified urethral catheters (AC5306CH06, Porges S.A., France) were inserted into the teat canal after a thorough disinfection of the teat orifice with 70% ethanol. Through the catheter, each mammary quarter was injected with 20 mL of PBS (0.01 M, pH 7.4; NaCL 0.138 M; KCL 0.0027 M, prepared with apyrogenic water) and lavages were immediately collected back through the catheter directly to the syringe. Immediately after harvesting resident cells, the mammary glands were treated with PBS (n = 20) or LPS (n = 20; 10 μg of LPS from *Escherichia coli *serotype O128:B12, Sigma, St. Louis, MO, USA) to induce an inflammatory response [25]. Inflammatory cell samples were obtained through lavage 24-168 h after administration of the PBS or LPS. These samples were identified as inflammatory macrophages (_INF_MAC).

### Cell processing and in vitro cultivation

Bacteriological examinations of all lavages were performed by culture on blood agar plates (5% washed ram erythrocytes) with aerobic incubation at 37°C for 24 h in duplicates. Only animals with sterile cultivation findings were included into the experiment. Total mammary cell counts in lavages were determined using the Fossomatic 90 apparatus (Foss Electric, Denmark) and the procedure recommended by the International Dairy Federation [26]. Cell suspensions were centrifuged at 4°C and 200 × g for 10 min. One milliliter of supernatant was retained for resuspension of the pellet. The remaining supernatant was decanted. All populations of macrophages were adjusted (5 × 10^6 ^cells/mL) in an RPMI 1640 medium (Sigma, MO, USA). A part of these cells was immediately analyzed (as a fresh cell population), and the remainder was incubated *in vitro*. Macrophages were put into microplates (6 × 4 Costar Ultraplates, CA, USA) and were incubated for 0, 3 and 6 h at 37°C. Following the incubation periods, incubated cells were analyzed using FCM. LDH was determined in the incubation medium.

### Flow cytometry

**Figure 1:**
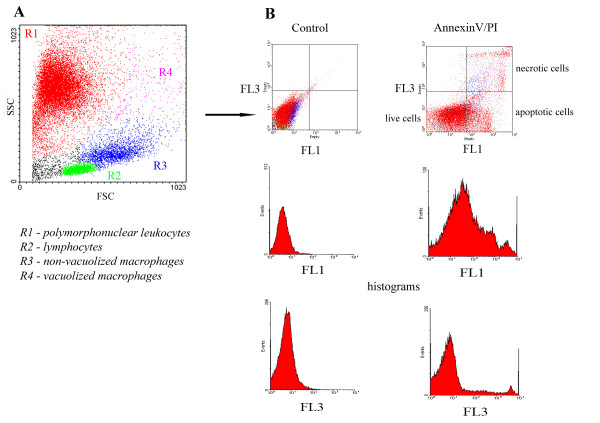
**The flow cytometry analysis of the cells from mammary glands**. The representative dot plot (A) shows the distribution of cells differentiated by their forward scatter and side scatter parameters. Next dot plots (B) and their histograms demonstrate Annexin V positivity (FL1 axis) and propidium iodide positivity (FL3) in control sample and in sample obtained 24 hour after intramammary instillation of PBS.

Each sample designated for FCM analysis was divided into two parts for detecting viability (apoptosis and necrosis) and for detecting CD14 and CD11b expression. The differential cell count was processed according to Sladek et al. [25] based on light scatter properties (Fig. 1). Apoptotic and necrotic macrophages were analyzed by FCM after being simultaneously stained with Annexin-V labeled with FITC and PI as described by Vermes et al. [27]. The commercial Annexin-V-FLUOS Staining Kit (Boehringer Mannheim, Mannheim, Germany) was used according to the manufacturer's instructions. For detecting CD14 by FCM, mouse anti-ovine CD14 (VPM65, Serotec, Oxford, UK) diluted 1:20 and PE-labeled swine anti-mouse IgG1 (SouthernBiotech, Birmingham, AL, USA), diluted 1:360, were used as the primary and secondary antibodies, respectively [25]. For detecting CD11b, MM10A (VMRD Inc., Pullman, WA, USA) diluted 1:20 and FITC labeled IgG2b (SouthernBiotech, Birmingham, Alabama, USA) diluted 1:100 were used as the primary and secondary antibodies, respectively. Negative control samples for AnnexinV and PI were not stained. Negative control samples for CD14 and CD11b were stained with the secondary antibody only. For analysis, we used the FACS Calibur flow cytometer with CELLQuest™ software (Becton Dickinson, Mountain View, CA, USA). The instrument setting for FCM was set to analyze 20,000 cells per sample.

### *In vitro *detection of cytolysis

The Cytotoxicity Detection Kit (Roche Diagnostic GmbH, Panzberg, Germany) was used to quantify mammary leukocyte cytolysis under the procedure used previously [28].

### Statistical analysis

Total cell count values were transformed by logarithmic transformation. All experimental characteristics - the cell counts (in logarithmic transformation), concentrations and the proportions - were tested for normal data distribution using the Shapiro-Wilk test. To determine significant sources of variability, the results were analyzed by multifactorial analysis of variance. The significance of differences in _RES_MAC and _INF_MAC before and during the inflammatory response and during *in vitro *cultivation were tested by determining the proportions of apoptotic and necrotic cells, CD14^+ ^and CD11b^+ ^cells, and LDH concentrations. These parameters were tested using Scheffé's method. Statistical analyses were carried out using STAT Plus software [29].

## Results

### Resident and inflammatory macrophages before and during inflammatory response

**Figure 2:**
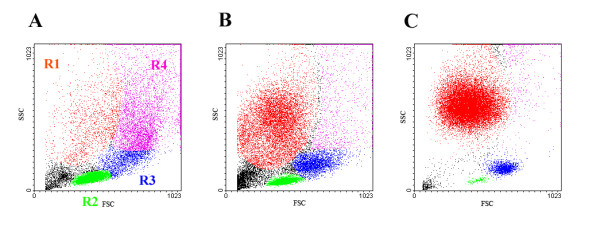
**The flow cytometry analysis of resident and inflammatory leukocytes from mammary glands**. Region distribution in dot plots of leukocytes from untreated heifer mammary glands (A) and leukocytes obtained 24 hour after intramammary instillation of PBS (B) and LPS (C) : polymorphonuclear leukocytes region (R1), lymphocyte region (R2), monocyte-like macrophages region (R3), and vacuolised macrophage region (R4).

The untreated mammary glands contained a resident cell population with relatively low total cell counts (0.6 ± 0.3 × 10^6^/mL). These cells were mainly comprised of _RES_MAC (53.8 ± 11.2%) and lymphocytes (36.1 ± 12.6%), and much less of PMN (10.1 ± 7.2%) (Fig. 2). In the _RES_MAC population, monocyte-like cells and a large number of vacuolized cells were observed (for a detailed structure and ultrastructure see Sladek and Rysanek [17]). Expression of integrin receptor CD11b on the surfaces of these cells was very low, at 27.9 ± 5.5% in monocyte-like _RES_MAC and 18.7 ± 2.1% in vacuolized _RES_MAC.

Intramammary application of PBS or LPS induced the inflammatory response, which was characterized by an influx of inflammatory cells. The total inflammatory cells count culminated at 24 h after treatment, and it was significantly higher following LPS than after PBS during all time points after treatment (*P *< 0.01, except *P *< 0.05 168 h) (Tab. 1).

In the initial stage of the inflammatory response (24 h), PMN comprised the dominant cell type in proportions of more than 50% and 90%, respectively, after PBS and LPS treatments (Fig. 2). In this time we determined only fleeting clinical signs (mild pain and moderate swelling) in examined animals, and particularly after LPS intramammary administration.

On the other hand, the resolution stage of the inflammatory response was characterized by the decrease in the PMN proportion (48 - 168 h). The proportion of macrophages increased at the same time, and 168 h after treatment it was very similar to that of the untreated mammary gland (data not shown).

**Figure 3:**
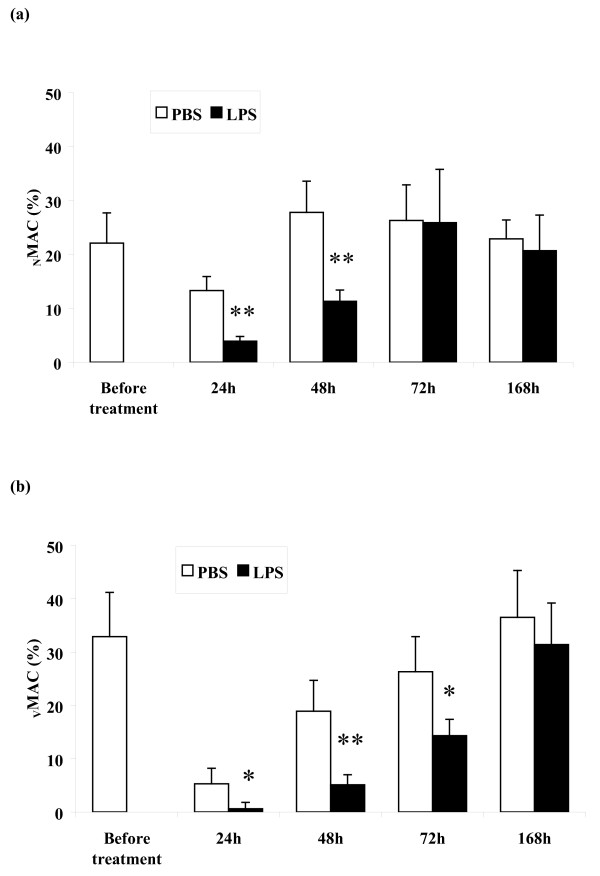
**Differential proportion of macrophages**. Differential proportion of non-vacuolized (a) and vacuolized macrophages (b) (mean ± S.D.) in mammary lavages collected before and at 24, 48, 72, and 168 h after intramammary instillation of PBS or LPS. Significant between-treatment differences are marked with asterisks (*P < 0.05; **P < 0.01).

Similarly to _RES_MAC from untreated mammary glands, _INF_MAC were also represented by two morphologically distinct types: monocyte-like cells and vacuolized cells with phagocytosed apoptotic PMN. The proportions of both types of macrophages in populations of _RES_MAC and _INF_MAC before and during the inflammatory response are shown in Fig. 3a, b.

In contrast to _RES_MAC, the expression of integrin receptor CD11b was significantly greater (*P *< 0.01) on the surface of monocyte-like and vacuolized _INF_MAC after PBS (64.4 ± 12.4% and 93.7 ± 1.7%) and after LPS (75.1 ± 13.1% and 99.5 ± 0.5%).

### Apoptosis and necrosis of resident macrophages from untreated heifer mammary glands

**Figure 4:**
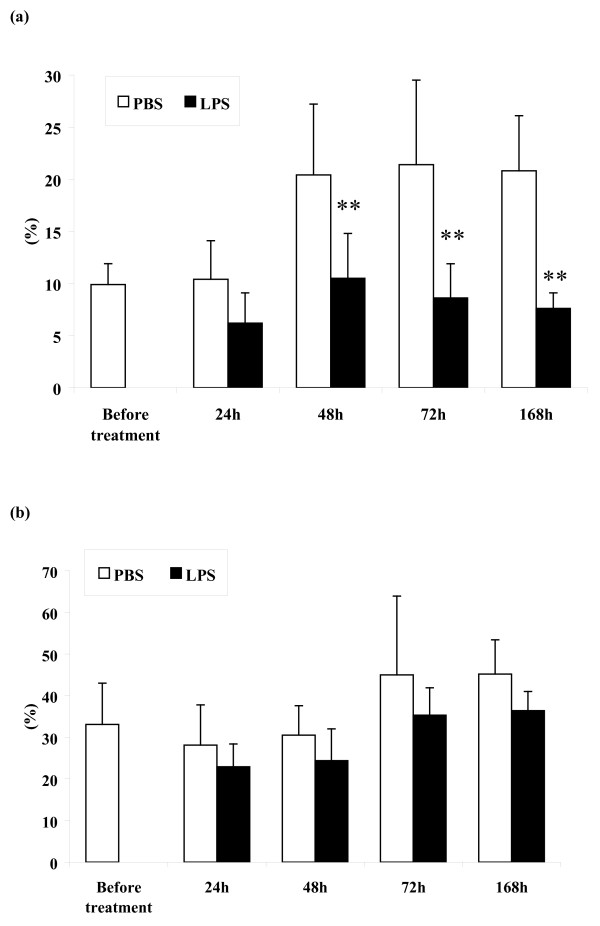
**Proportion of apoptotic macrophages *in situ***. Relative proportion of apoptotic cells in population of non-vacuolized (a) and vacuolized macrophages (b) (mean ± S.D.) in mammary lavages collected before and 24, 48, 72, and 168 h after intramammary instillation of PBS or LPS. Significant between-treatment differences are marked with asterisks (**P < 0.01).

Apoptotic and necrotic cells were differentially detected in both morphologically different types of _RES_MAC. In the _RES_MAC population approximately one-tenth of monocyte-like cells and one-third of vacuolized cells were apoptotic (Fig. 4a, b). Necrosis was observed in 7% of monocyte-like _RES_MAC and in 23% of vacuolized _RES_MAC.

### Apoptosis and necrosis of inflammatory macrophages during the inflammatory response

In the _INF_MAC population obtained 24 h after PBS treatment, approximately one-tenth of monocyte-like cells and almost one-quarter of vacuolized cells were apoptotic (Fig. 4a, b). At the same time after LPS treatment, however, we observed a insignificantly lower proportion of apoptotic cells in the population of monocyte-like _INF_MAC and vacuolized _INF_MAC (Fig. 4a, b).

Moreover, a higher proportion of apoptotic cells in populations of monocyte-like _INF_MAC and vacuolized _INF_MAC was detected during all time points after PBS in contrast to LPS. As is evident from Fig. 4a, statistically significant differences between proportions of apoptotic cells were observed for 48-168 h (*P *< 0.01) in monocyte-like _INF_MAC while no significant differences existed for vacuolized _INF_MAC. Furthermore, when comparing _RES_MAC and _INF_MAC, we observed that vacuolized _RES_MAC and vacuolized _INF_MAC underwent apoptosis more intensively than did monocyte-like _RES_MAC and monocyte-like _INF_MAC.

In the population of _INF_MAC obtained 24-168 h after PBS treatment, fewer than 5% of monocyte-like cells and fewer than 20% of vacuolized cells were necrotic. In addition, we observed an insignificantly higher proportion of necrotic cells in the population of monocyte-like _INF_MAC 24-168 h after LPS treatment. In the population of vacuolized _INF_MAC, we also detected a higher proportion of necrotic cells after LPS than PBS treatment (except at the time point 168 h). Differences between the proportions of necrotic cells were at no time significant during the experimental period.

### Apoptosis and necrosis of resident and inflammatory macrophages in vitro

*In vitro *cultivation of _RES_MAC and _INF_MAC led to great changes in the proportions of apoptotic and necrotic cells.

**Figure 5:**
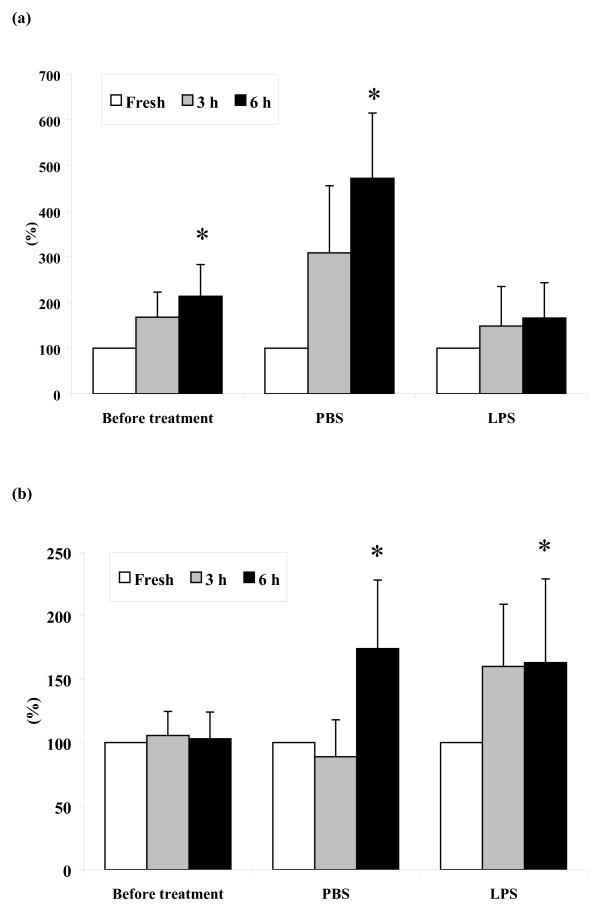
**Proportion of apoptotic macrophages *in vitro***. Relative proportion of apoptotic cells in population of non-vacuolized (a) and vacuolized macrophages (b) obtained before and 24 h after intramammary instillation of PBS or LPS during *in vitro *cultivation (mean ± S.D.). Significant between-treatment differences are marked with asterisks (*P < 0.05).

**Figure 6:**
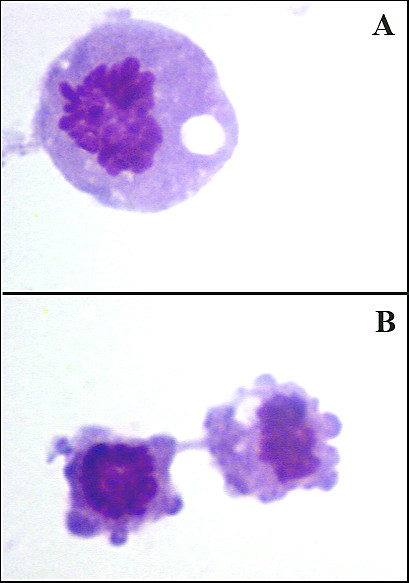
**Apoptosis of macrophages after cultivation *in vitro *in light microscopy**. Light microscopy of macrophages cultivated *in vitro *for 6 h showing morphological features of apoptosis, such as fragmentation and condensation of the chromatin, vacuolization (**A**), and membrane blebbing (**B**). May-Grünwald Giemsa stain (Pappenheim method). Magnification: 1000× (A) and 800× (B).

We observed after *in vitro *cultivation of _RES_MAC that the proportion of apoptotic cells was significantly increased only in monocyte-like cells (*P *< 0.05) (Fig. 5a). After *in vitro *cultivation of _INF_MAC, on the other hand, apoptosis was significantly increased in monocyte-like cells only after PBS (*P *< 0.05) and in vacuolized cells after PBS (*P *< 0.05) and LPS (*P *< 0.05) (Fig. 5a, b). The morphological features of apoptosis in monocyte-like _RES_MAC during *in vitro *cultivation are shown in Fig. 6.

Similarly to apoptosis, the proportions of necrotic cells were significantly increased in all populations of _RES_MAC (except monocyte-like cells) and _INF_MAC during *in vitro *cultivation (data not shown).

### Effect of incubation time on cell loss

**Figure 7:**
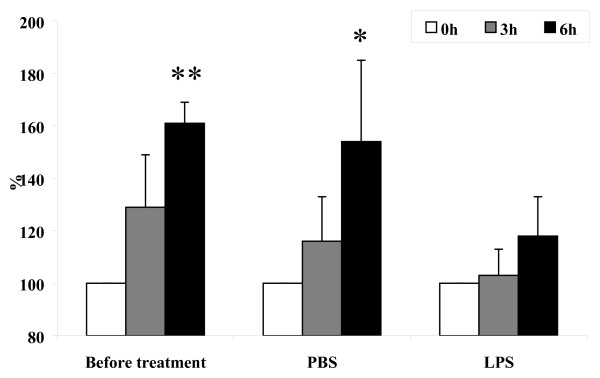
**Detection of LDH during cultivation *in vitro***. Detection of LDH during *in vitro *cultivation of resident macrophages obtained from untreated mammary gland and inflammatory macrophages obtained 24 h after intramammary instillation of PBS or LPS (mean ± S.D.). Significant between-treatment differences are marked with asterisks (*P < 0.05; **P < 0.01).

*In vitro *cultivation was also accompanied by an increase of LDH in all populations of macrophages (Fig. 7), indicating cell loss. The highest level of cytolysis was detected in _RES_MAC as compared to _INF_MAC, although the cytolysis of _INF_MAC following treatment with PBS was higher than after LPS. These results indicate a significantly higher cell loss in the _RES_MAC population in comparison with that of _INF_MAC during *in vitro *cultivation.

### CD14 expression in resident and inflammatory macrophages in vitro

**Figure 8:**
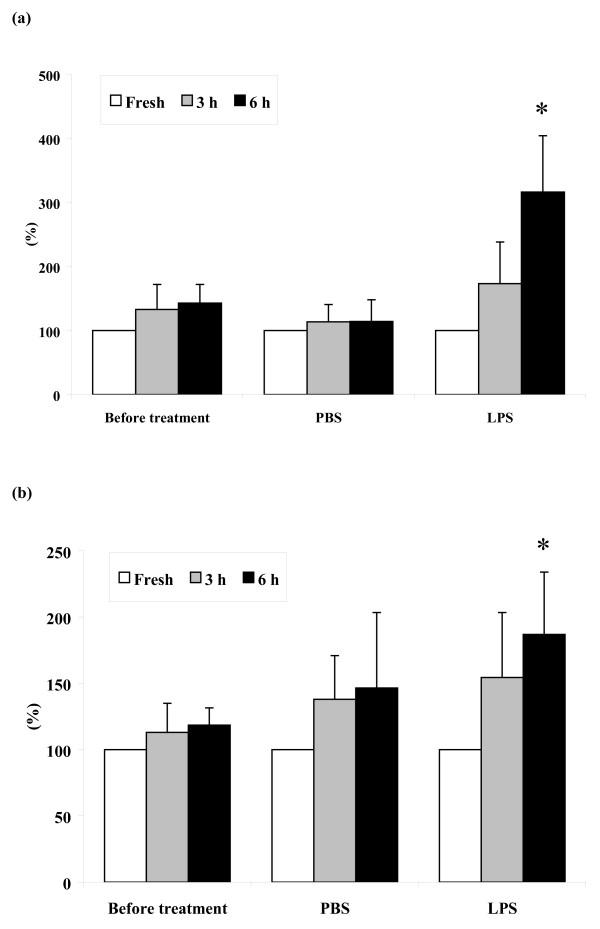
**Expression of CD14 in macrophages**. Relative proportion of CD14 positive cells in population of non-vacuolized macrophages (a) and vacuolized macrophages (b) obtained before and 24 h after intramammary instillation of PBS or LPS (mean ± S.D.). Significant between-treatment differences are marked with asterisks (*P < 0.05).

*In vitro *cultivation was accompanied by a differential activation of _RES_MAC and _INF_MAC, which was evident from the change in the proportion of CD14^+ ^cells. As demonstrated in Fig. 8a, b, the proportion of CD14^+ ^cells was increased in all populations of macrophages during *in vitro *cultivation. However, significant differences were observed only in the populations of monocyte-like _INF_MAC (*P *< 0.01) and vacuolized _INF_MAC (*P *< 0.05) obtained 24 h after LPS treatment.

## Discussion

The aim of this study was to confirm the occurrence of apoptosis in resident and inflammatory macrophages from heifer mammary glands, as no data is available on this subject in veterinary medicine. In this pilot study, an inflammation model based on inducing inflammatory response in mammary glands of virgin heifers was used.

The mammary gland of heifers is characterized by the presence of three distinct populations of resident cells: macrophages, lymphocytes and PMN. Previously, it had been observed that PMN and lymphocytes may undergo apoptosis. Moreover, it had been established that apoptosis of these cells plays an important role during the inflammatory response of mammary glands [9,11,23,30]. In this study, we determined that macrophages of heifer mammary glands also undergo apoptosis. To our knowledge, this is the first study that dealing with apoptosis of macrophages from bovine mammary glands.

_RES_MAC from heifer mammary glands comprise the dominant cell population, which consists of two morphologically different cell types. They are sometimes categorized as monocytes (non-vacuolized, monocyte-like cells) and numerous (vacuolized) macrophages [3,22,25,31]. This morphological categorization of macrophages from bovine mammary glands is important. As the two types of macrophages have different biological features [25,31], we expected different proportions of apoptosis and necrosis in the two populations of macrophages.

In this study, we observed approximately 10% apoptotic and less than 8% necrotic cells in the fresh population of monocyte-like _RES_MAC. This low proportion of death cells is to be expected, because monocyte-like _RES_MAC are relatively young cells in comparison to vacuolized _RES_MAC. It has been suggested that monocyte-like _RES_MAC are derived from surrounding tissues or blood monocytes [3,32]. Migration of these cells can be a constitutive process that occurs at a much lower level in the absence of any apparent cue [33]. After migration into tissues, these monocyte-like cells undergo further differentiation to become multifunctional tissue macrophages with fully developed scavenger function. Apoptotic cell death is, however, required in order for maintain homeostasis [34], and the detection of apoptosis in these cells suggests that monocyte-like _RES_MAC are not resistant to apoptosis. Moreover, when we cultured these cells *in vitro*, the proportion of apoptotic cells was significantly increased. This seems to be normal, as it has been shown that monocytes and/or macrophages cultured *in vitro *without any stimulus become apoptotic within less than 24 h [35].

The relatively low number of apoptotic monocyte-like _RES_MAC suggests that the major parts of these cells survive, monitoring inflammatory or other danger signals, and phagocytosing the cellular material of sloughed epithelial and other cells from ducts of mammary glands, as we found in our previous study [17]. As a consequence of their scavenger function, monocyte-like _RES_MAC may change into vacuolized forms approximately 5 and more days after migration [3]. In the population of vacuolized _RES_MAC in this study, however, we observed a higher proportion of apoptotic cells (33.1%). A very similar situation seems to be observed in the human lung. In the alveolar microenvironment of the healthy human lung, alveolar macrophages have been shown to have a high apoptotic rate (62.1%), since apoptotic cell death is required for homeostasis and lung architecture to be maintained [34]. In contrast to monocyte-like _RES_MAC, the proportion of apoptotic cells was not surprisingly increased after *in vitro *culturing. This suggests that part of apoptotic vacuolized _RES_MAC may consequently undergo secondary necrosis [36] and therefore the proportion of necrotic cells was significantly increased together with LDH concentration during *in vitro *cultivation, as we have observed recently for a PMN population [28]. Cytolysis of these cells has biological significance in the fact that they released chemotactic factors initiating influx of inflammatory cells.

The intramammary instillation of PBS or LPS resulted in an inflammatory response with a massive influx of PMN during the initial stage. In addition, macrophages also migrated from the blood as monocytes through the surrounding tissues into the mammary gland [25,37]. In contrast to _RES_MAC, therefore, these freshly migrated cells expressed high levels of CD11b adhesion receptor [38]. Moreover, the inflammatory forms of macrophages possess monocyte-like morphology and represent a dominant type of macrophages during the initial phase of the inflammatory response [22]. During resolution, however, these cells are vacuolized due to their scavenger function and became numerous during this time. As PMN underwent apoptosis and were subsequently phagocytosed by _INF_MAC during resolution of the inflammatory response [9,10], it is evident that, similarly to _RES_MAC, two morphologically different cells also exist in the _INF_MAC population: monocyte-like cells and vacuolized cells. In comparison to vacuolized _RES_MAC, however, the vacuolized _INF_MAC contain phagocytosed apoptotic PMN in their cytoplasm, as was described in our previous studies [9,30,39].

In this study, we observed approximately 10% apoptotic and less than 3% necrotic cells in the fresh population of monocyte-like _INF_MAC 24 h after PBS treatment and only 5% apoptotic cells after LPS treatment. Similarly to the situation of monocyte-like _RES_MAC, this low proportion of death cells is to be expected, because monocyte-like _INF_MAC are relatively young cells derived from monocytes and are rescued from early apoptotic death [40]. Furthermore, we observed differences between treatments in the proportions of apoptotic monocyte-like _INF_MAC during the entire experimental period. We assume that after LPS treatment the macrophage apoptosis is halted by inflammatory stimuli that prolong their survival. Since LPS is the most potent factor that rescues monocytes from apoptosis by inducing autocrine synthesis of the inflammatory cytokines, tumor necrosis factor alpha (TNF-α) and interleukin-1 (IL-1) [41], these cytokines in particular have been detected at increased levels during the initial stage of inflammation caused by *Escherichia coli *[42]. When we cultured monocyte-like_INF_MAC *in vitro*, the proportion of apoptotic cells was significantly increased only after PBS, in contrast to LPS. Furthermore, a significant increase in expression of CD14 surface receptor on these cells was recorded after LPS during *in vitro *cultivation. CD14 is a very important macrophage/monocyte surface molecule shown to induce activation in response to LPS. The aforementioned TNF-α and IL-1 also increase CD14 expression and enhance monocyte survival [43,44]. It is suggested, therefore, that LPS may play an important role in regulating apoptosis not only in PMN [10] but also in macrophages from bovine mammary glands.

Nevertheless, the proportion of apoptotic monocyte-like _INF_MAC was increased by more than twice during resolution of the inflammatory responses caused by PBS and LPS. The literature now contains good evidence that _INF_MAC can undergo apoptosis at the inflamed site even in cases of sterile inflammation. Inflammation caused by noninfective challengers may lead to inducible nitric oxide synthase synthesis and hence high levels of nitric oxide. Nitric oxide is well known as a trigger for macrophage apoptosis [45]. Serum deprivation can also lead to apoptosis in macrophages, albeit at a much lower level than in other cells [46]. It seems that induction of apoptosis in _INF_MAC is a physiological and altruistic mechanism that may help to reduce inflammatory stress and to avoid the establishment of chronic persistent inflammatory response. In contrast, however, apoptosis of macrophages has also been observed in pathological processes. Recently, it was revealed that induction of macrophage apoptosis and subsequent secondary necrosis is caused by bacterial exotoxin with histotoxic effect [47]. The proapoptotic effect of *Mycobacterium tuberculosis *on lung macrophages and the contradictory pathogenic effect are also known [48].

Furthermore, we observed that apoptosis in vacuolized _INF_MAC was higher than in monocyte-like _INF_MAC during the entire experimental period and without significant differences between treatments. Surprisingly, we observed no effect of LPS treatment on delay of apoptosis in vacuolized _INF_MAC during *in vitro *cultivation. It is known that phagocytosis of apoptotic PMN is associated with apoptosis of macrophages. Local apoptosis of macrophages once having ingested apoptotic cells has been observed during inflammation [40]. In this case, the clearance of these macrophages also seems to be an important manner for successfully resolving inflammation.

## Conclusion

In conclusion, our experiments confirmed that macrophages of the heifer mammary glands also undergo apoptosis. We conclude that apoptosis of _RES_MAC is accompanied by natural senescence. On the other hand, apoptosis of _INF_MAC seems to be one of the important events in resolution of the inflammatory response. In addition, secondary necrosis of apoptotic macrophages and cytolysis was determined. However, the exact role of apoptosis in macrophages from bovine mammary glands, and particularly in host-pathogen interactions, needs to be elucidated in future studies.

## Competing interests

The authors declare that they have no competing interests.

## Authors' contributions

ZS carried out the practical work, performed flow cytometry analysis, compiled the results, participated in interpretation of results, drafted the manuscript and participated in its revision. DR was mainly responsible for performing the statistical analyses, helped in interpreting the results and revising the manuscript. Both authors read and approved the final manuscript.
